# Anti-inflammatory Effects of Heme Oxygenase-1 Depend on Adenosine A_2A_- and A_2B_-Receptor Signaling in Acute Pulmonary Inflammation

**DOI:** 10.3389/fimmu.2017.01874

**Published:** 2017-12-20

**Authors:** Franziska M. Konrad, Constantin Zwergel, Kristian-Christos Ngamsri, Jörg Reutershan

**Affiliations:** ^1^Department of Anesthesiology and Intensive Care Medicine, University Hospital of Tübingen, Tübingen, Germany; ^2^Department of Anesthesiology and Intensive Care Medicine, Hospital of Bayreuth, Bayreuth, Germany

**Keywords:** heme oxygenase-1, pulmonary inflammation, neutrophil, PMN, hemin

## Abstract

Acute pulmonary inflammation is still a frightening complication in intensive care units. In our previous study, we determined that heme oxygenase (HO)-1 had anti-inflammatory effects in pulmonary inflammation. Recent literature has emphasized a link between HO-1 and the nucleotide adenosine. Since adenosine A_2A_- and A_2B_-receptors play a pivotal role in pulmonary inflammation, we investigated their link to the enzyme HO-1. In a murine model of pulmonary inflammation, the activation of HO-1 by hemin significantly decreased polymorphonuclear leukocyte (PMN) migration into the lung. This anti-inflammatory reduction of PMN migration was abolished in A_2A_- and A_2B_-knockout mice. Administration of hemin significantly reduced chemokine levels in the BAL of wild-type animals but had no effects in A_2A^-/-^_ and A_2B^-/-^_ mice. Microvascular permeability was significantly attenuated in HO-1-stimulated wild-type mice, but not in A_2A^-/-^_ and A_2B^-/-^_ mice. The activity of HO-1 rose after LPS inhalation in wild-type animals and, surprisingly, also in A_2A^-/-^_ and A_2B^-/-^_ mice after the additional administration of hemin. Immunofluorescence images of animals revealed alveolar macrophages to be the major source of HO-1 activity in both knockout strains—in contrast to wild-type animals, where HO-1 was also significantly augmented in the lung tissue. *In vitro* studies on PMN migration further confirmed our *in vivo* findings. In conclusion, we linked the anti-inflammatory effects of HO-1 to functional A_2A_/A_2B_-receptor signaling under conditions of pulmonary inflammation. Our findings may explain why targeting HO-1 in acute pulmonary inflammation has failed to prove effective in some patients, since septic patients have altered adenosine receptor expression.

## Introduction

Acute pulmonary inflammation and its more severe form acute respiratory distress syndrome, are life-threatening complications in intensive care units (ICU) with a high mortality of 40% (1). Surviving patients have a decreased quality of life (2). Acute pulmonary inflammation can be caused either by direct lung injury, such as pneumonia, or indirect injury, such as sepsis or trauma. These different etiological factors might be the reason why, despite decades of research, no single treatment has been successful and the translation of animal models in clinical practice has failed (3). Therefore, newer studies focus on subgroups of patients because individualized treatments may be successful (3).

Polymorphonuclear leukocytes (PMNs) are part of the innate immune system and are the first cells to migrate toward inflammation. In the case of acute pulmonary inflammation, PMNs excessively migrate from the intravascular compartment through an endothelial barrier into the lung interstitium, followed by a transepithelial migration into the alveolar space. These migration steps underlay different regulation (4) and destroy the lung architecture.

Adenosine triphosphate is released in response to cellular stress and is extracellularly degraded to adenosine diphosphate by the membrane-bound nucleotidase CD39, followed by further degradation by CD73 to adenosine (5). Adenosine, a purine nucleoside, acts *via* the four adenosine receptors (A_1_, A_2A_, A_2B_, and A_3_). All of these adenosine receptors have been shown to be involved in inflammation (6–9); specifically, the A_2A_- and A_2B_-receptors play a crucial role in acute pulmonary inflammation (10).

The A_2B_-receptor, for example, exerts a protective role in ischemic lung injury (11), and an inhaled A_2B_-receptor agonist has been recommended for the treatment of acute lung injury (12).

Heme oxygenase (HO)-1 is a ubiquitous enzyme in the body that catalyzes the degradation of heme. HO-1 is pharmacologically inducible and known as a potent cytoprotective enzyme that is upregulated under different conditions of cellular stress (13). Although the stimulation of HO-1 in acute pulmonary inflammation has been shown to have anti-inflammatory effects in several animal studies (14, 15), clinical trials have so far failed to be convincing, showing only a trend toward a reduction in inflammatory cells in the treatment of chronic obstructive disease (COPD) or even having no effect on endotoxemia-induced inflammation (16, 17). Therefore, current research on HO-1 and its products demands further study of the function of the HO-1 enzyme in the inflammatory setting since this remains complex and still incompletely understood (18).

Recent literature suggests a link between HO-1 and adenosine receptors (19–21). Adenosine receptors play a pivotal role concerning the two hallmarks of pulmonary inflammation: PMN migration and microvascular permeability (8, 9). Therefore, we thought to investigate the link between the anti-inflammatory effects of HO-1 and the predominant pulmonary adenosine receptors A_2A_ and A_2B_ to potentially identify subgroups of patients where a specific stimulation of HO-1 would be a therapeutic option. Patients in ICUs sometimes have altered adenosine receptor distribution and ligand affinities (22).

## Materials and Methods

### Animals

We used A_2A_ (A_2A^-/-^_) and A_2B_ gene-deficient mice (A_2B^-/-^_) (from Dr. Katya Ravid, Boston University, School of Medicine, Department of Biochemistry, Boston, MA, USA) and obtained corresponding wild-type mice (CD1 and C57BL/6 respectively) from Charles River Laboratories (Germany). Mice were males and between 8 and 12 weeks old. All animal protocols were approved by the Animal Care and Use Committee of the University of Tübingen.

### HO-1 Activator and -Inhibitor

Hemin was injected intraperitoneally (i.p.) (80 µmol/kg) 24 h before LPS inhalation to increase HO-1 activity (15). To inhibit the enzyme, SnPP (50 µmol/kg) was additionally administered i.p. 1 h before LPS inhalation (15). The specific equilibrative nucleoside transporter 1 (ENT1) antagonist NBTI was administered as described previously (9). Cobalt (III) protoporphyrin-IX-chloride (CoPP) was injected at 5 µg/g body weight i.p. 24 h before the inflammatory stimulus (23).

### HO-1- Expression and Protein Level

Total RNA was isolated from murine lung samples by using pegGOLD TriFast™ (Peglab, Germany), and cDNA synthesis was performed by Bio-Rad iSkript-kit (Bio-Rad, Germany) according to the manufacturer’s direction.

We determined the expression of HO-1 in the lungs of wild-type, A_2A^-/-^_, and A_2B^-/-^_ mice by RT-PCR (*n* = 5–8). This method was performed with the following HO-1 primers (5′-GAG ATT GAG CGC AAC AAG GA-3′ and AGC GGT AGA GCT GCT TGA ACT-3′) as described (9). 18S was used as house keeping gene (5′-GTA ACC CGT TGA ACC CCA TT-3′ and 5′-CCA TCC AAT CGG TAG TAG CG-3′).

To further verify the gene expression results on a protein level, we measured HO-1 protein expression in the lungs of mice by ELISA (ENZO Life Sciences, Lörrach, Germany) (*n* = 5–8). Mice inhaled LPS and HO-1 was induced, respectively, inhibited as described above.

### HO-1 Activity

Lungs of mice were removed to measure the enzymatic activity of HO-1 24 h after LPS inhalation (*n* = 6–8). HO-1 was induced by hemin, respectively, inhibited by the additional administration of SnPP, lungs were removed and weighed and HO-1 activity buffer (twice of weight) added. The samples were frozen in liquid nitrogen. Lungs were homogenized, sonicated, and centrifugated at 18,000 *g* for 15 min. The supernatant was used for protein- and activity-measurement. Cytosol of the liver was obtained from 12 h fastened mice *via* centrifugation at 105,000 *g* for 27 min. The HO-1 activity assay consisted of 100 µl of the supernatant, 131 µl of HO-activity buffer, 100 µl of liver cytosol, 50 µl of glucose-6-phosphate (20 nM), and 10 µl of hemin (1 mM). After incubating for 1 h in the dark at 37°C, 500 µl chloroform was added, followed by a centrifugation of 15,000 *g* for 5 min. Extinction was measured at 464 and 530 nm, and HO-activity was calculated based on protein level, which was determined by a colorimetric method (bicinchoninic acid; Thermo Scientific, Rockford, IL, USA).

### Immunofluorescence Images

Heme oxygenase-1 was stimulated as described above, inflammation induced and lungs of mice removed after 24 h (*n* = 4). The circulatory system of the lungs was flushed and lungs with 4% paraformaldehyde (PFA) for 10 min at 25 cmH_2_O inflated. Lungs were removed and fixed in PFA for 24 h. Rabbit polyclonal anti-HO-1 was used as primary antibody (Enzo, Life Sciences GmbH, Lörrach, Germany) to mark the enzyme and the endothelial marker von Willebrand Factor (Santa Cruz Biotechnology, Santa Cruz, CA, USA) was employed and additionally DAPI for nuclear staining. Images were visualized by using a confocal microscope (LSM 510, Meta, Carl Zeiss). Images shown are representatives of four experiments and were analyzed using ImageJ, a public program developed at the National Institutes of Health to officially analyze scientific images.

### *In Vivo* Migration Assay

#### Murine Model of Acute Lung Injury

Four to eight animals inhaled nebulized LPS as described in detail before (24). This inhalation caused acute pulmonary inflammation with a reproducible migration of PMNs into the different compartments of the lung (intravascular, interstitial, intraalveolar), an increase of chemokines, and microvascular permeability (15, 25). Control mice inhaled the solvent.

#### *In Vivo* Migration Assay

24 h after LPS inhalation, we determined PMN migration into the different compartments of the lung *via* a flowcytometry-based method as described in detail before (*n* = 5–9) (24). To mark all intravascular PMNs, a fluorescent GR-1 (clone RB6-8C5) was injected into the tail vein of mice. Lungs were flushed free of blood by injecting saline into the beating right ventricle, to remove nonadherent leukocytes from the pulmonary vasculature. PMNs from the alveolar space were determined in the BAL. Lungs were incubated with fluorescent antibodies to CD45 (clone 30-F11) and 7/4 (clone 7/4). Intravascular PMNs were now identified as CD45+, 7/4+, and GR-1+; whereas interstitial PMNs were assigned as CD45+, 7/4+, and GR-1 negative cells. Absolute cell counts were determined in the BAL and lungs. The detailed description of the method and the gating process has been described elsewhere (24).

### Depletion of Ectonucleotidases CD39 and CD73

SiRNA of CD39 and CD73 (Santa Cruz Biotechnology, Heidelberg, Germany) was used to knock down gene expression of both ectonucleotidases in pulmonary epithelial cells (NCI-H441). In H441 cells, HO-1 was activated by hemin (5 µM), and cells were stimulated with LPS (100 ng/ml) for 4 h. Gene expression of interleukin-6 (IL-6) and 8 (IL8) were detected in cells by RT-PCR (*n* ≥ 5) by using the following primers: IL6 (5′-GAC AGC CAC TCA CCT CTT CA-3′ and 5′-CAC CAG GCA AGT CTC CTC AT-3′); IL8 (5′-TGT GGG TCT GTT GTA GGG-3′ and 5′-GTG AGG TAA GAT GGT GGC-3′); and 18S as house keeping gene (5′-GTA ACC CGT TGA ACC CCA TT-3′ and 5′-CCA TCC AAT CGG TAG TAG CG-3′).

Interleukin-6 and IL8 protein were detected in the supernatant of cells by ELISA (R&D Systems, Minneapolis, MN, USA) (*n* ≥ 5).

### Chemokine Release

3 h after LPS inhalation, the release of CXCL1, CXCL2/3, tumor necrosis factor-α (TNFα), and IL-6 was measured in the BAL of mice (*n* = 5–9) by ELISA (R&D) according to the manufacture’s protocol.

### Microvascular Leakage

Evans blue extravasation was determined as a marker of capillary leakage as described before (9). 6 h after LPS exposure, Evans blue (20 mg/kg, Sigma-Aldrich, Steinheim, Germany) was injected into the tail vein (*n* = 5–8). Intravascular Evans blue in the lungs was removed 30 min later by flushing the beating right ventricle. Lungs were homogenized and Evans blue was extracted with formamide and the final concentration determined colorimetrically (26).

### *In Vitro* PMN Migration

We performed the *in vitro* transmigration assay of human PMNs through a monolayer of NCI-H441 cells (*n* = 6–12) or primary pulmonary endothelial cells (HMVEC-L, Lonza Walkersville, MD, USA) (*n* = 6–10) as described previously (9). HO-1 was induced by hemin (5 µM) and the A_2A_- (ZM 241385; Tocris, MO, USA) (10 ng/ml) or A_2B_-receptors (PSB 1115 Sigma-Aldrich, Taufkirchen, Germany) (10 ng/ml) were inhibited with the respective antagonists.

### Statistical Analysis

Data are presented as the mean ± SD unless indicated otherwise. Statistical analysis was performed using GraphPad Prism version 5.3 for Windows (GraphPad Software, San Diego, CA, USA). Differences between the groups were evaluated by one-way ANOVA followed by Bonferroni *post hoc* test or Student *t*-Test to compare two groups. *P* < 0.05 was considered statistically significant.

## Results

### Gene and Protein Expression of HO-1 in the Lungs of Mice

**Figure 1:**
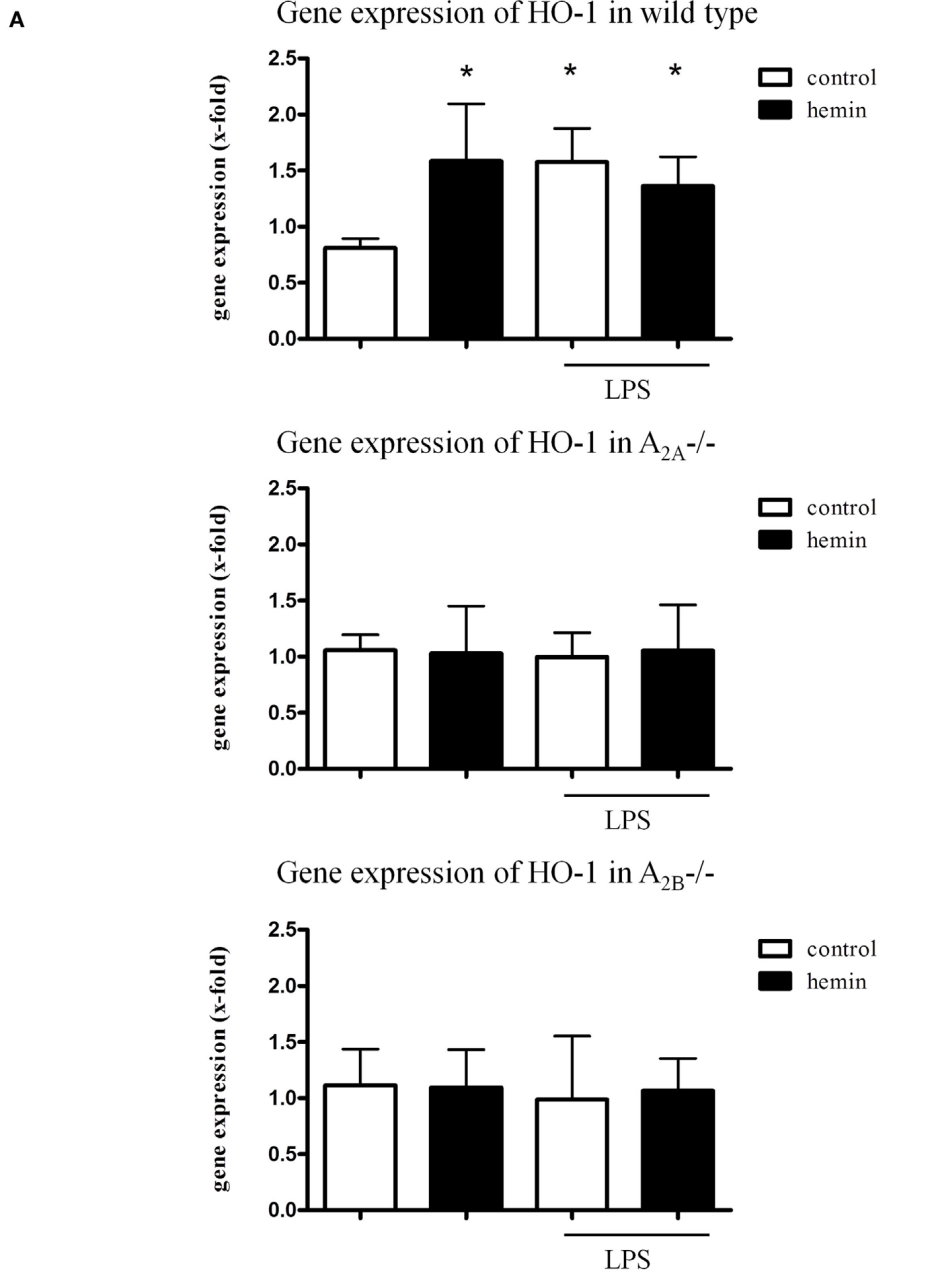

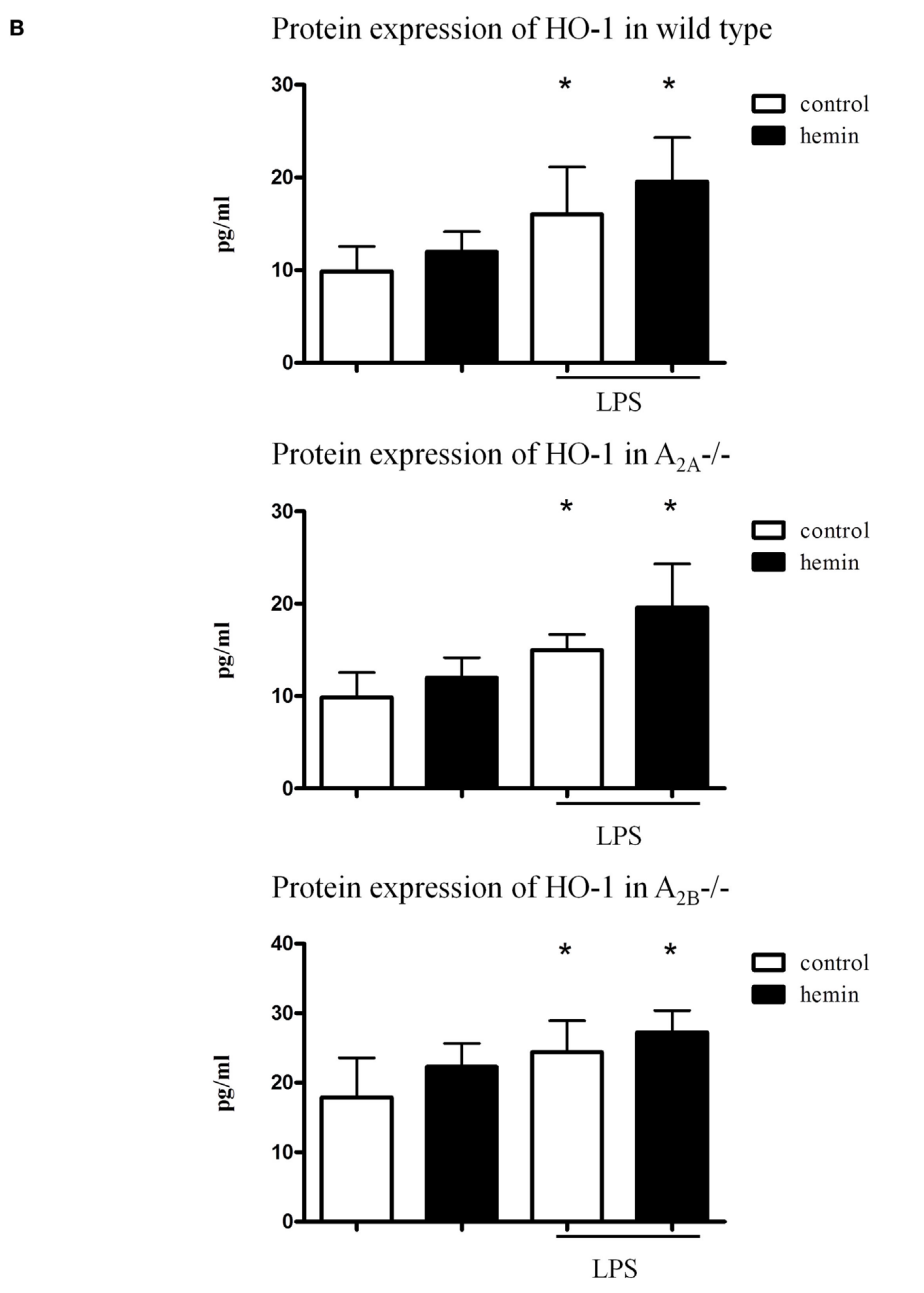

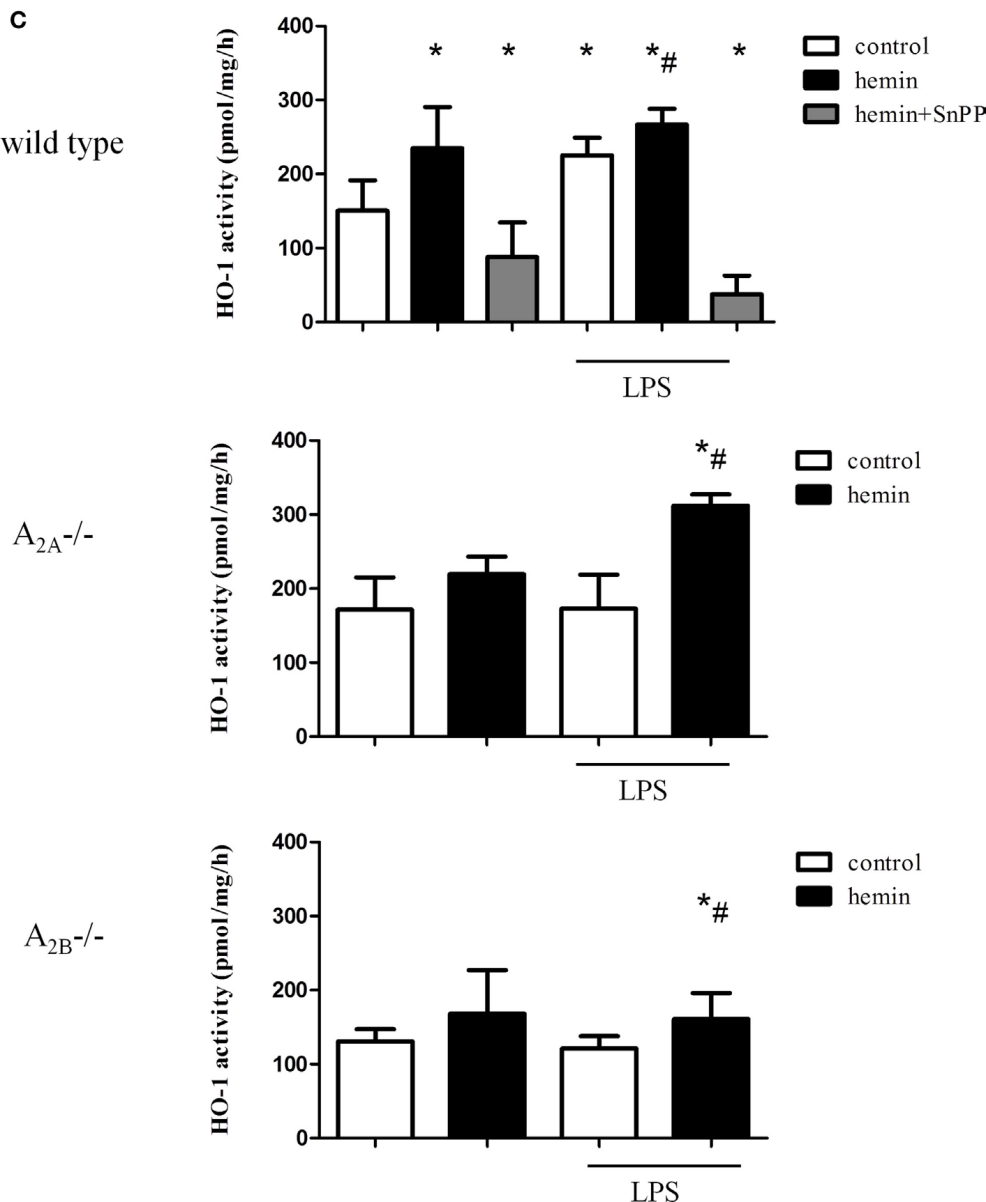
Heme oxygenase (HO)-1 gene and protein expression and activity in the lungs of mice. Gene **(A)** and protein **(B)** expression of HO-1 in the lungs of mice was determined. The activity of the enzyme was evaluated after the activation of HO-1 by hemin and the inhibition by additional administration of SnPP **(C)**. Data are presented as the mean ± SD; without LPS inhalation, *n* = 4–8 other groups, *n* = 5–8; *P < 0.05 vs. control without LPS; ^#^*P* < 0.05 vs. LPS-treated mice.

In wild-type mice, hemin caused a significant increase in the gene expression of HO-1 in the lungs even without inflammation (all *P* < 0.05). Since both wild-type mice acted identically, we display only one strain in Figure 1 (CD1 in Figure 1A C57BL/6 in Figure S1 in Supplementary Material). LPS also caused a significant rise, and the additional HO-1 stimulation did not show an additive effect on the gene expression.

Neither inflammation nor HO-1 activation by hemin changed the expression of HO-1 in A_2A^-/-^_ and A_2B^-/-^_ mice.

Protein expression of HO-1 in the lungs of mice confirmed the significant increase in the enzyme in LPS-treated wild-type animals (all *P* < 0.05) (Figure 1B; Figure S1B in Supplementary Material). Surprisingly, the augmentation after inflammation was also apparent in A_2A^-/-^_ and A_2B^-/-^_ mice at the protein level.

### Influence of A_2A^-/-^_ and A_2B^-/-^_ Receptor Signaling on the Activity of HO-1

To further verify our findings from the gene and protein expression experiments, we measured the activity of the HO-1 enzyme. In wild-type animals, hemin increased HO-1 activity without inflammation, whereas the inhibition caused a significant reduction (all *P* < 0.05) (Figure 1C; Figure S1C in Supplementary Material). Inflammation also enhanced HO-1 activity, and the additional activation of the enzyme showed an additive effect.

In A_2A^-/-^_ and A_2B^-/-^_ mice, hemin did not influence the activity of HO-1 without inflammation. Additionally, LPS did not change the activity, but additional hemin administration caused a significant increase in HO-1 activity in both strains (all *P* < 0.05).

### Induction of HO-1 in Alveolar Macrophages and Lung Tissue

**Figure 2:**
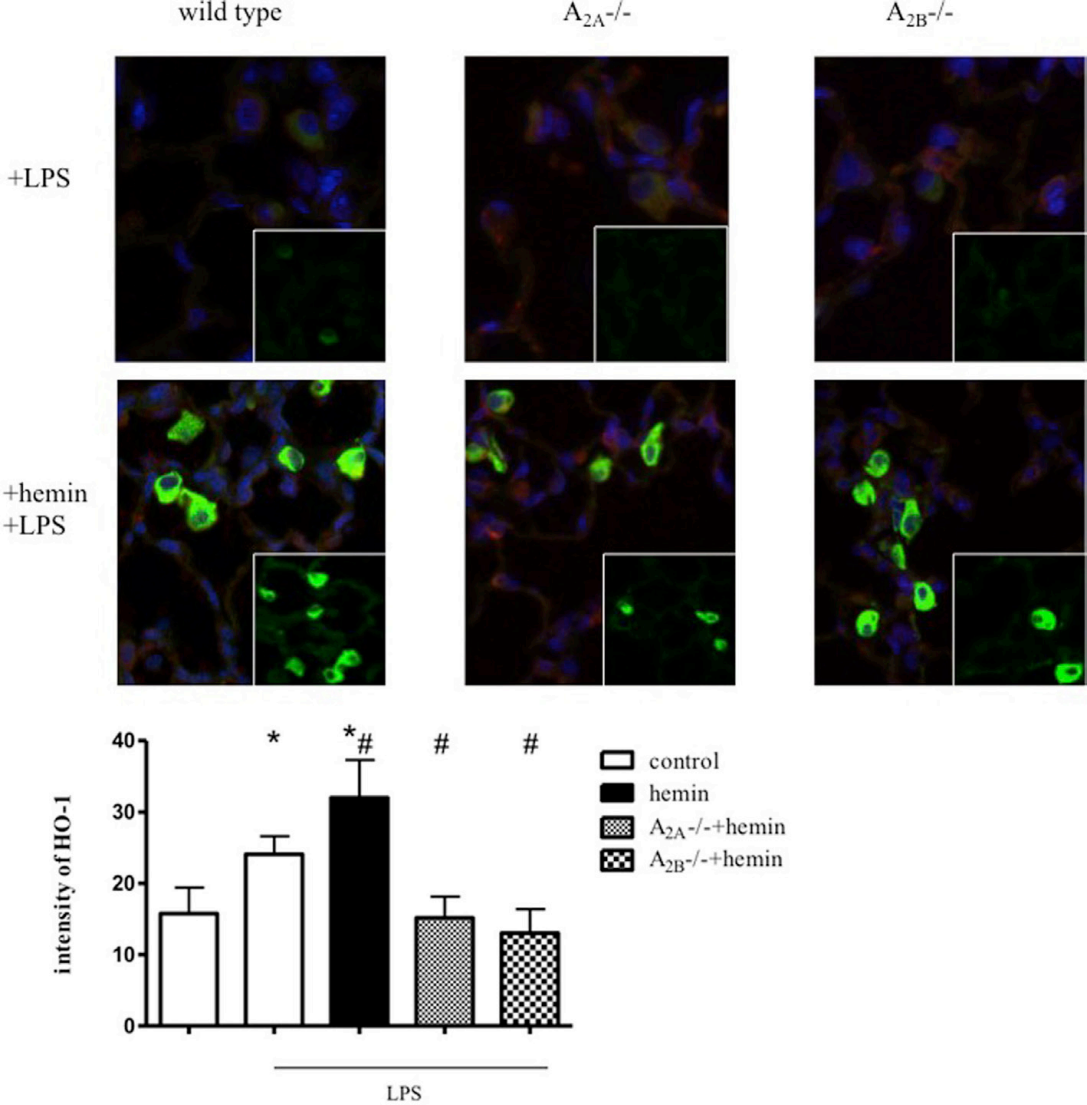
Determination of heme oxygenase (HO)-1 expression by immunofluorescence. Mice were treated with LPS, and HO-1 was induced by hemin in wild-type, A_2A^-/-^_, and A_2B^-/-^_ mice (*n* = 4). Images are representatives of four experiments with similar results (original magnification, 63×). HO-1 was stained with a specific antibody and appears green, nuclei were stained with DAPI and emerge blue, cytokeratin appears red. Intensity was measured only in the lung tissue, excluding alveolar macrophages. Data are presented as the mean ± SD; *n* = 6; *P < 0.05 vs. control; ^#^*P* < 0.05 vs. LPS-treated mice.

To evaluate the unclear source of HO-1 activity, we performed immunofluorescence staining of lung sections. Without inflammation, HO-1 was only slightly increased in wild-type animals after hemin treatment (Figure S2 in Supplementary Material). Inflammation significantly increased the expression of HO-1 in lung tissue (*P* < 0.05) (Figure 2). In wild-type animals, hemin further increased HO-1 in lung tissue and alveolar macrophages (*P* < 0.05).

In A_2A^-/-^_ and A_2B^-/-^_ mice, inflammation and the administration of hemin did not change HO-1 expression in lung tissue. The activation of HO-1 increased HO-1 expression in alveolar macrophages in these mice, explaining our findings from the protein expression experiment and the determination of the activity of the enzyme. We had previously determined that HO-1 is expressed in pulmonary tissue, alveolar macrophages, and PMNs, but the anti-inflammatory effects of HO-1 in acute pulmonary inflammation are based on the expression of the enzyme in the tissue (15).

### Anti-inflammatory Effect of HO-1 on PMN Migration Depends on Adenosine Receptor Signaling

**Figure 3:**
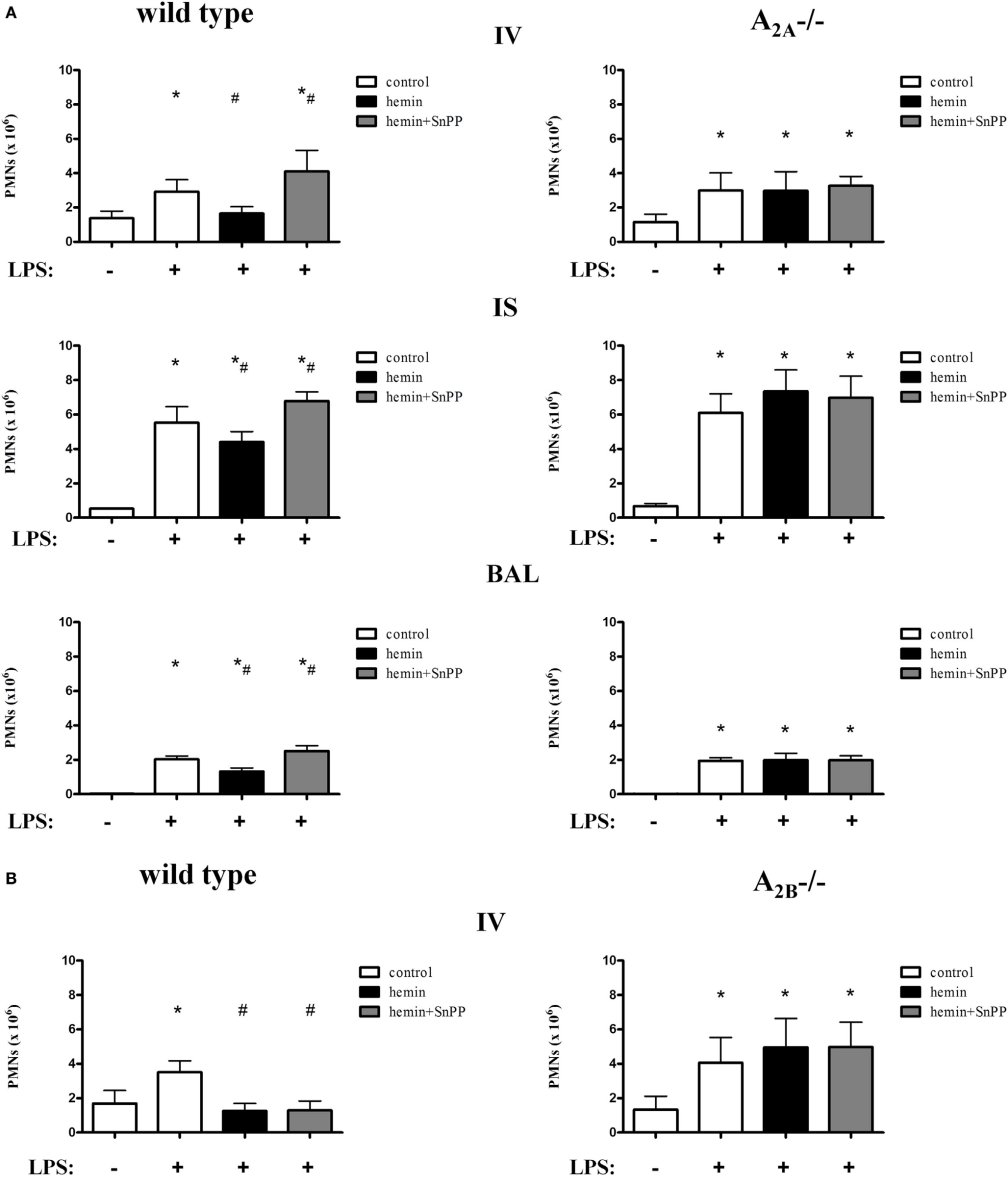

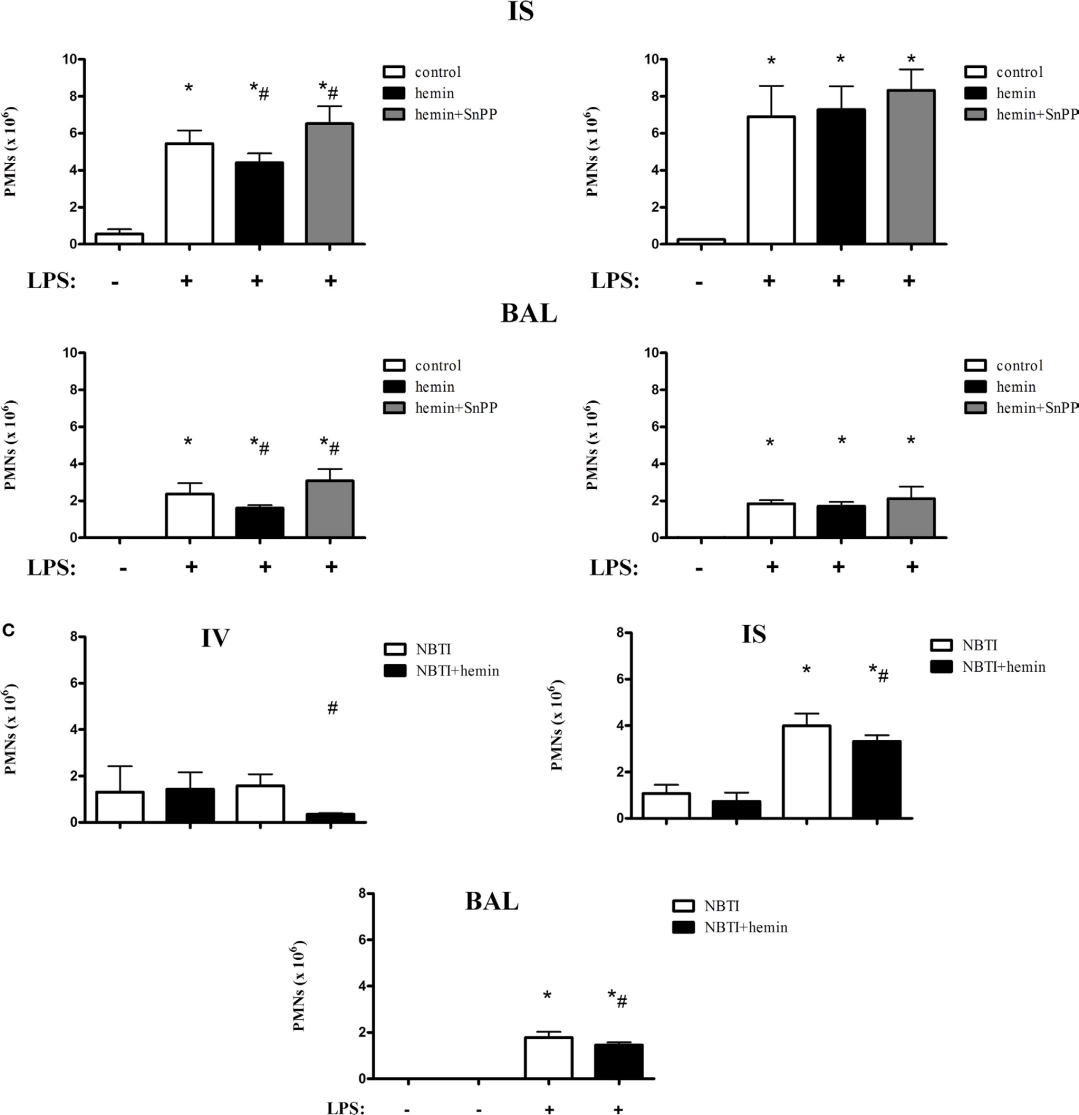

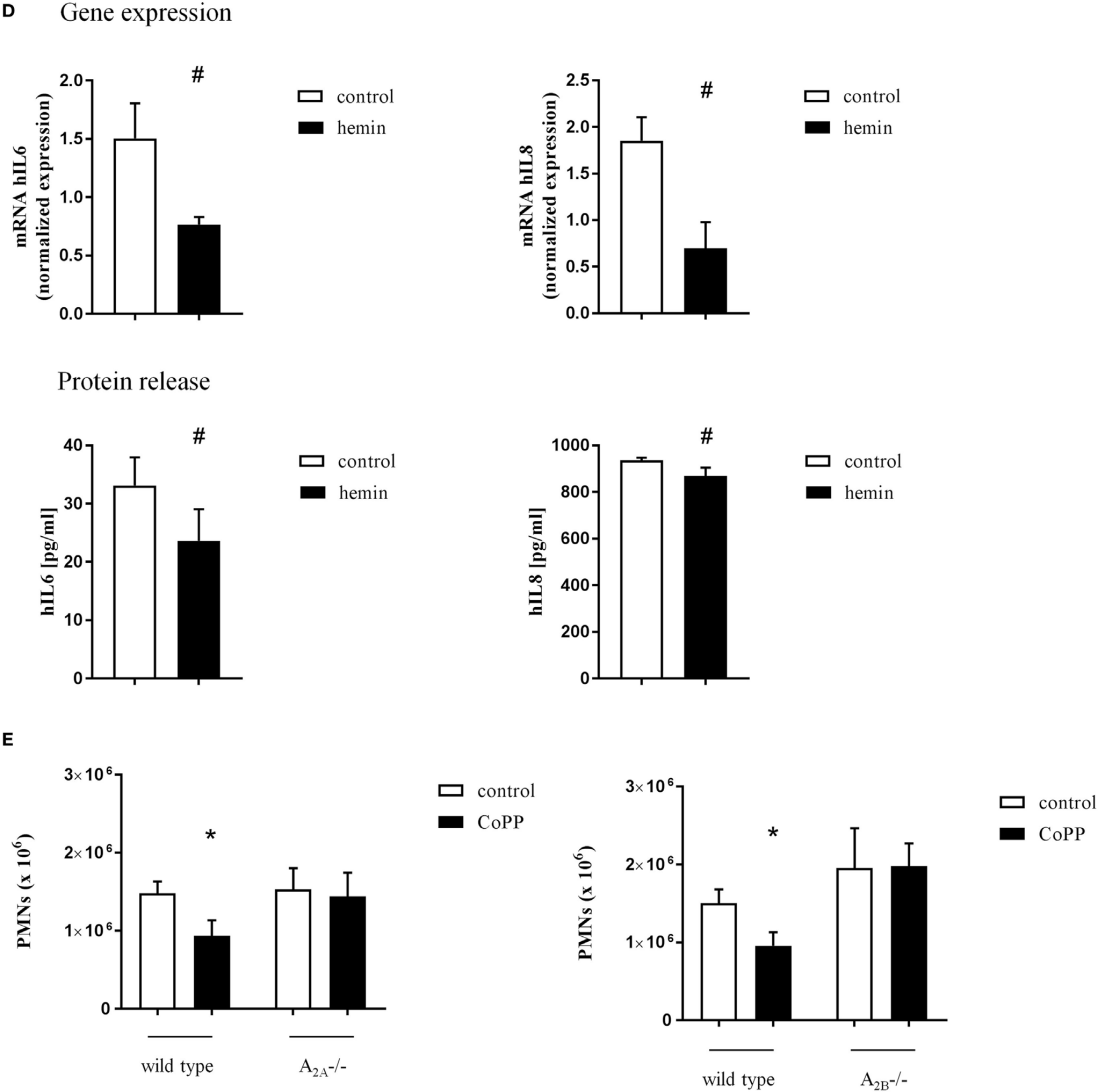
Migration of polymorphonuclear neutrophils (PMNs) into the different compartments of the lung after activation of heme oxygenase (HO)-1. HO-1 was induced by hemin and inhibited by the additional administration of SnPP in wild-type, A_2A^-/-^_ **(A)**, and A_2B^-/-^_ mice **(B)**. Mice were treated with LPS, and the migration of PMNs into the different compartments of the lung (IV, intravascular space; IS, interstitial space; BAL, bronchoalveolar space) was evaluated. The cellular uptake of adenosine was inhibited by the specific equilibrative nucleoside transporter 1 (ENT1) inhibitor NBTI and the effect of HO-1 induction by hemin on PMN migration into the different compartments of the lung was evaluated **(C)**. Extracellular adenosine levels were reduced by the depletion of the ectonucleotidases CD39 and CD73 in human pulmonary epithelial cells. The impact of HO-1 stimulation and inflammation with LPS was investigated by the determination of human interleukin 6 and 8 in these cells **(D)** by RT-PCR. Protein concentrations of both chemokines were detected in the supernatant. PMN counts were determined after LPS inhalation in the BAL of wild-type, A_2A^-/-^_, and A_2B^-/-^_ mice after HO-1 stimulation by CoPP **(E)** (*n* = 7–8). Data are presented as the mean ± SD; without LPS inhalation, *n* = 4 other groups, *n* = 5–9; *P < 0.05 vs. control without LPS; ^#^*P* < 0.05 vs. LPS-treated mice.

In wild-type animals, stimulation or inhibition of HO-1 did not influence PMN counts in animals without inflammation (Figure S3 in Supplementary Material). LPS inhalation significantly increased the migration of PMNs into the intravascular, interstitial, and alveolar space, whereas the stimulation of HO-1 by hemin significantly reduced the PMN influx into all three compartments (all *P* < 0.05) (Figures 3A,B). Inhibition of HO-1 increased the attachment of PMNs to the endothelium and significantly enhanced PMN migration into the lung interstitium and alveolar space.

Without inflammation, neither stimulation nor inhibition of the enzyme influenced the number of PMNs in the lungs of A_2A^-/-^_ mice (Figure S3A in Supplementary Material). LPS caused a significant increase in PMNs in the intravascular, interstitial, and intra-alveolar space (all *P* < 0.05) (Figure 3A). In contrast to wild-type animals, stimulation and inhibition of HO-1 did not affect PMN counts in any compartment of the lung.

Next, we investigated the impact of the adenosine receptor A_2B_ on the enzyme HO-1 in this setting (Figure S3B in Supplementary Material). Similar to the wild-type animals of the A_2A^-/-^_ mice, the wild-type animals of these knockout mice showed a reduction in the number of PMNs in all three compartments of the lung in response to HO-1 stimulation (Figure 3B). Inhibition of the enzyme significantly reversed this effect in the lung interstitial and alveolar space (all *P* < 0.05). In A_2B^-/-^_ mice, neither activation nor inhibition of the enzyme affected PMN counts in any of the lung compartments, highlighting the importance of A_2A_- and A_2B_-receptor signaling in the anti-inflammatory effects of HO-1.

As previously shown by our group, A_2A^-/-^_ and A_2B^-/-^_ mice have an augmented inflammatory reaction to LPS inhalation compared to wild-type animals (8, 27). In the present study, we did not directly compare wild type, A_2A^-/-^_ and A_2B^-/-^_ mice. The experimental setting was rather based on the identification of the effects of stimulating HO-1 in these mice. Therefore, the composition of the study groups has been shown to affect the results (9, 28).

### Anti-inflammatory Effects of HO-1 Do Not Depend on Extracellular Adenosine Levels

Elevation of extracellular adenosine has anti-inflammatory effects in acute inflammation (29). To investigate if the anti-inflammatory effects of hemin are mainly based on changes in extracellular adenosine levels or are specifically mediated by A_2A_-/A_2B_-receptor signaling, we performed additional experiments. The ENT1 is a transmembrane protein that mediates the cellular uptake of adenosine (30). We increased the extracellular levels of adenosine by the specific ENT1 antagonist NBTI and investigated the therapeutic effect of hemin. There were no differences in PMN migration into the lung between the groups without LPS (Figure 3C). After inflammation, the PMN counts were significantly increased in the lung interstitium and the alveolar space and were significantly reduced by the administration of hemin (all *P* < 0.05), suggesting that the anti-inflammatory effects of hemin are not based on increased adenosine uptake in our model.

To further prove the A_2A_- and A_2B_- dependency of the anti-inflammatory effects of HO-1, we investigated the impact of lower extracellular adenosine levels on the anti-inflammatory effects of the enzyme. CD39 and CD73 are the enzymes that convert precursor nucleotides to adenosine; therefore, the knockdown of both enzymes decreases extracellular adenosine detrimental. In our experiments, hemin still exhibited anti-inflammatory effects concerning the expression and the release of IL6 and IL8 after the knockdown of both ectonucleotidases in human pulmonary epithelial cells (Figure 3D). These results confirm that hemin has still anti-inflammatory effects at reduced adenosine levels.

### Anti-inflammatory Effects of the Enzyme HO-1 Depend on A_2A_- and A_2B_-Receptor Signaling Independent from the HO-1 Stimulus

CoPP, as an alternative to hemin, was used to stimulate HO-1. These separate experiments were performed to exclude that the observed effects of mice lacking the A_2A_-, respectively, the A_2B_-receptor are specific for HO-1 induction by hemin. All mice inhaled LPS and PMN migration into the alveolar space was evaluated (Figure 3E). In both wild-type mice, the administration of CoPP decreased migrated PMNs into the alveolar space as described before (23). In A_2A^-/-^_ and A_2B^-/-^_ mice, the administration of CoPP did not affect PMN counts, concordantly to our results using hemin for HO-1 stimulation.

### HO-1-Reduced Chemokine Release Is Linked to A_2A_- and A_2B_-Receptor Signaling

**Figure 4:**
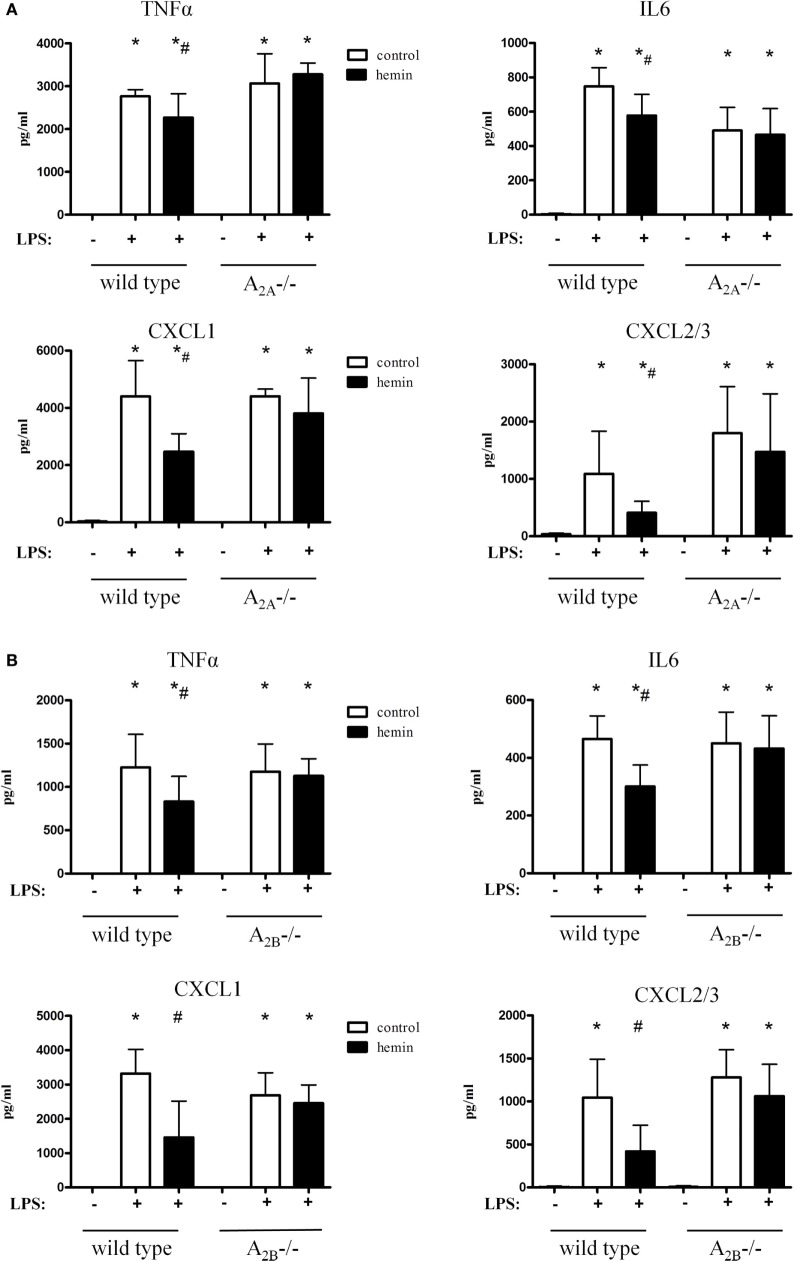
Chemokine release in the bronchoalveolar lavage fluid of mice after activation of heme oxygenase (HO)-1. HO-1 was induced by hemin in wild-type, A_2A^-/-^_ **(A)**, and A_2B^-/-^_ mice **(B)**. Levels of TNFα, IL6, and the most PMN chemoattractive chemokines CXCL1 and CXCL2/3 were determined in the BAL of mice. Data are presented as the mean ± SD; without LPS inhalation, *n* = 4 other groups, *n* = 5–9; *P < 0.05 vs. control without LPS; ^#^*P* < 0.05 vs. LPS-treated mice.

Inhalation of LPS significantly increased TNFα, IL6 and the most potent chemoattractive chemokines for PMNs, CXCL1, and CXL2/3 (all *P* < 0.05). Hemin reduced all four chemokine levels in both wild-type strains (Figures 4A,B), confirming our results from the *in vivo* PMN migration assay.

In A_2A^-/-^_ animals, the inflammation caused a significant rise of all chemokines (Figure 4A). Activation of HO-1 did not affect the release of chemokines in these mice, thereby further verifying our findings from the *in vivo* transmigration assay.

Hemin did not affect the chemokine levels in A_2B^-/-^_ mice, highlighting and further emphasizing the A_2A_- and A_2B_-dependent anti-inflammatory effect of HO-1 (Figure 4B).

### HO-1-Reduced Microvascular Permeability Depends on A_2A_- and A_2B_-Receptor Signaling

**Figure 5:**
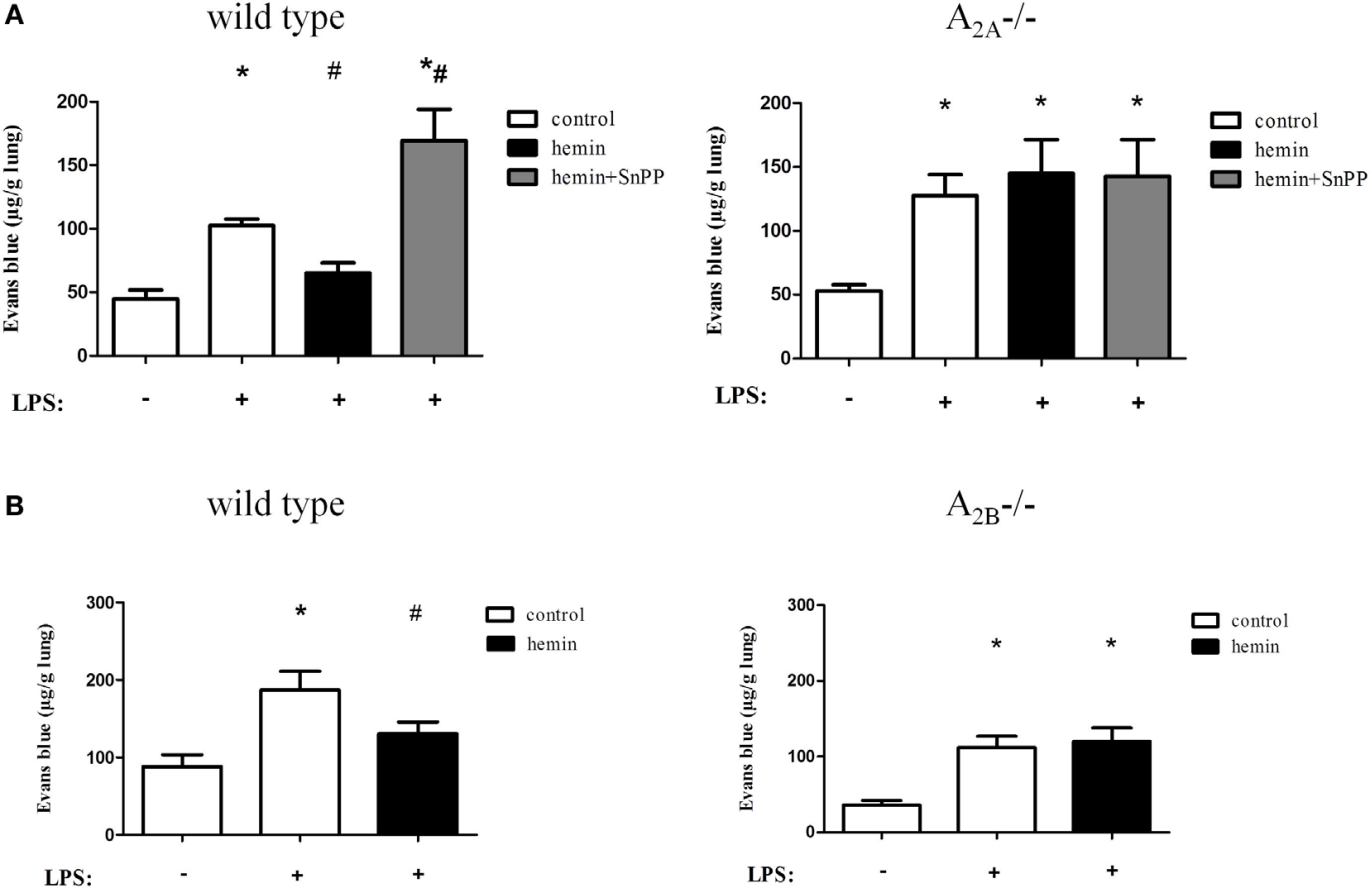
Anti-inflammatory effects of heme oxygenase (HO)-1 on microvascular permeability. Capillary leakage was assessed by Evans blue extravasation, and the influence of HO-1 in wild-type, A_2A^-/-^_ **(A)**, and A_2B^-/-^_ **(B)** mice was investigated. HO-1 was induced by hemin and inhibited by the additional administration of SnPP. Data are presented as the mean ± SD; without LPS inhalation, *n* ≥ 4 other groups, *n* = 5–8; *P < 0.05 vs. control without LPS; ^#^*P* < 0.05 vs. LPS-treated mice.

Extravasation of Evans blue as an indicator for the permeability of albumin and, therefore, a marker for capillary leakage was assessed (Figures 5A,B). Stimulation and inhibition of the enzyme HO-1 without inflammation did not affect microvascular permeability (Figure S4 in Supplementary Material). LPS inhalation increased Evans blue significantly, whereas the stimulation of HO-1 preserved capillary leakage almost to baseline (all *P* < 0.05) (Figure 5A). As a control, inhibition of the enzyme was shown to further increase the microvascular permeability in wild-type mice.

Inflammation increased the extravasation of Evans blue in A_2A^-/-^_ (Figure 5A) and A_2B^-/-^_ mice (Figure 5B). Activation and inhibition of HO-1 did not affect capillary leakage in A_2A^-/-^_ mice. We also demonstrated that HO-1 activation in A_2B^-/-^_ mice had no effect on microvascular permeability, highlighting the pivotal role of A_2A^-/-^_ and A_2B^-/-^_ receptor signaling in conjunction with the enzyme HO-1 in the appearance of the second hallmark of acute pulmonary inflammation.

### *In Vitro* PMN Migration Assay

**Figure 6:**
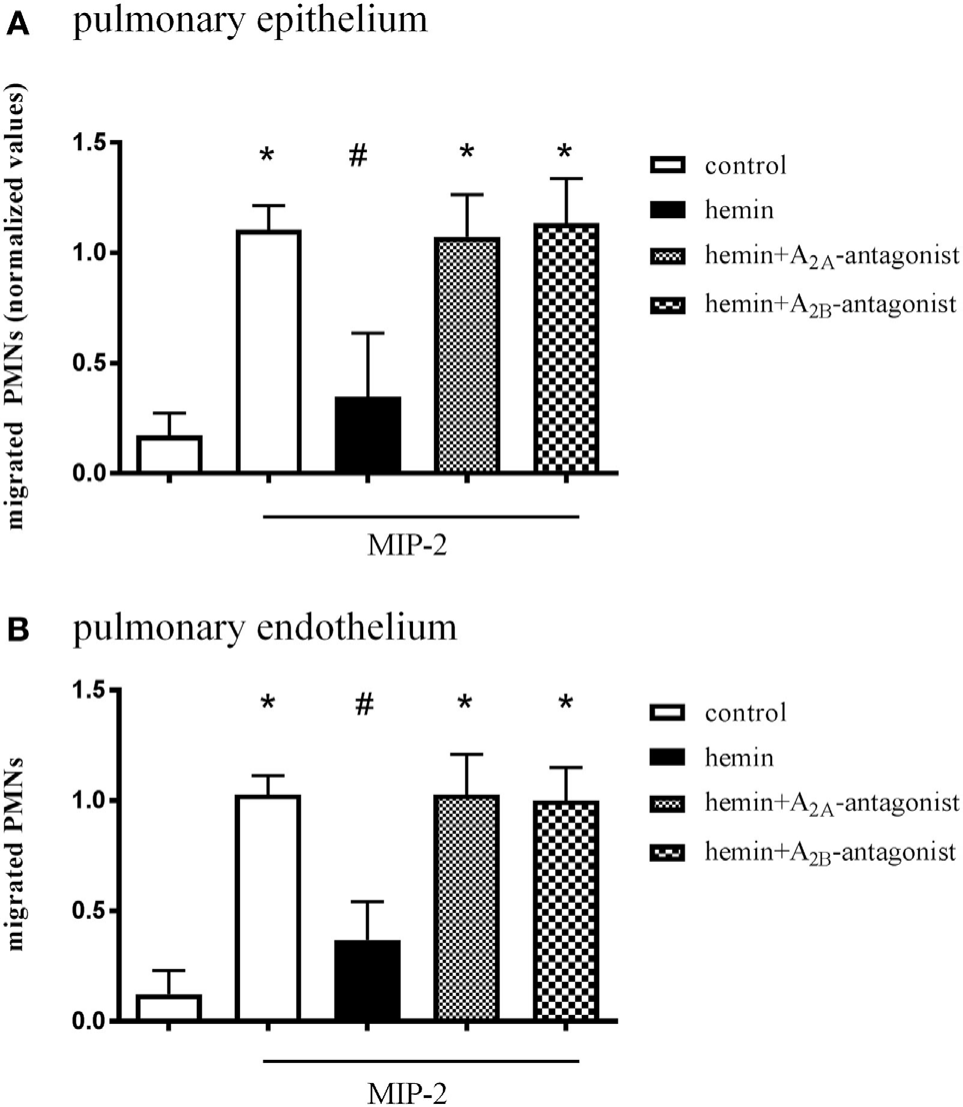
Effect of A_2A_/A_2B_-receptor signaling on pulmonary epithelium and endothelium. *In vitro* transmigration assay of human PMNs through a pulmonary epithelial **(A)** and endothelial **(B)** monolayer. Epithelium and endothelium were treated with hemin and specific A_2A_/A_2B_-receptor antagonists, and the migration of PMNs through a monolayer of human epithelium/endothelium was measured. The migration of PMNs was initiated *via* the chemokine MIP-2 (CXCL2/3) at indicated wells. Data are presented as the mean ± SD; *n* = 6–12; *P < 0.05 vs. control; ^#^*P* < 0.05 vs. MIP-2-treated wells.

In this assay, isolated human PMNs migrate through a monolayer of pulmonary epithelial/endothelial cells along a chemotactic gradient. Since the anti-inflammatory effect of HO-1 in the setting of acute pulmonary inflammation depends on the expression of the enzyme in lung tissue (15), the pulmonary epithelium/endothelium was treated with hemin and specific A_2A_/A_2B_-receptor antagonists. Hemin treatment of epithelial (Figure 6A) and endothelial cells (Figure 6B) significantly reduced the number of migrated PMNs (all *P* < 0.05). After treatment with the specific A_2A_/A_2B_-receptor antagonists, the stimulation of HO-1 was ineffective in reducing the PMN influx in both cell lines, confirming our *in vivo* experiments.

## Discussion

Gene polymorphism in the HO-1 gene promoter (HMOX1) has been shown to be associated with increased HO-1 levels and a reduced risk of acute pulmonary distress syndrome (31). HO-1, the inducible form of HO, is upregulated in tissue during cellular stress and influences inflammation in different settings (14, 32–35).

However, the mechanism by which HO-1 elicits this cytoprotection remains incompletely understood but might be a combination of the removal of heme (a pro-oxidant iron chelate) and the enzymatic generation of biologically active end products with anti-inflammatory effects (36). The end products include iron, biliverdin, and carbon monoxide (CO). CO, as an inhaled gas, has been used to induce HO-1 with anti-inflammatory effects in several studies including those on endotoxemia (37), ventilator-induced lung injury (38), and ischemia/reperfusion injury (39).

In the presented study, we used hemin to induce HO-1. In a previous study from our group, we demonstrated the impact of this agent on the anti-inflammatory effects in acute pulmonary inflammation in terms of PMN migration, microvascular permeability, chemokine release, oxidative burst, and the formation of stress fibers (15). Additionally, we highlighted the impact of the topical administration of hemin with a significantly lower required dose and simultaneously more anti-inflammatory effects. These observations were in accordance with the actual literature, where aerosol therapy in critical care medicine is considered fundamental (40) and there is convincing evidence that nebulized administration of agents elicits higher concentrations and, therefore, better efficiency than systemic application (41–43). Additionally, typical side effects of systemic hemin administration such as headaches, nausea, and vomiting might be less prominent using the topical administration (44). Furthermore, hemin has been verified to induce the expression of HO-1 in humans (44) and is already successfully in use for the treatment of acute intermittent porphyria (45). To ensure a clinically relevant study, we also used hemin for the induction of HO-1 in the presented study.

Here, we confirmed the anti-inflammatory effects of HO-1 in acute pulmonary inflammation in two different mouse strains. We used the common inbred strain C57BL/6, which are almost genetically homogenous, and the outbred CD1 mouse strain, which has a higher genetic diversity. CD1 mice are outbred mice with a complex genetic history, comparable to a human founder population (46). Recent literature on acute pulmonary inflammation notes the importance of genetic variation in this disease (47, 48). We, therefore, demonstrated the pivotal role of hemin as an inductor of HO-1 in a condition of acute pulmonary inflammation independent of slight genetic variations. By the additional inhibition of HO-1 in selected experiments, which resulted in aggravation of the inflammation, we verified that the detected results were based on the enzyme HO-1.

Adenosine receptor signaling plays a crucial anti-inflammatory role in inflammation by protecting tissue from inflammation-related damage (49). These receptors modulate chemokine production and secretion (50). Our data link the anti-inflammatory effects of HO-1 on PMN migration, chemokine release, and microvascular permeability to A_2A/2B_-signaling. Extracellular adenosine uptake plays a pivotal role in pulmonary inflammation (51). In the presented study, HO-1 still had anti-inflammatory effects after the specific inhibition of the cellular adenosine uptake by the transmembrane protein ENT1 and, therefore, an elevation of extracellular adenosine. HO-1 stimulation also exhibited anti-inflammatory effects at reduced adenosine levels, suggesting rather a direct effect of stimulating HO-1 on A_2A/2B_-receptors than an adenosine-dependent mechanism.

The link between the adenosine system and HO-1 is in line with the findings of Reichelt et al. (20). They investigated the influence of a nonspecific adenosine receptor agonist on the expression of HO-1 and the enzyme activity in ischemic hearts of mice. Weis et al. showed that downregulation of HO-1 resulted in a decreased expression of the anti-inflammatory A_2A_-receptor in macrophages (21). Additionally, adenosine was found to perceptibly induce HO-1 and attenuate LPS-induced TNFα levels *via* the A_2A_-receptor (19).

The expression of HO-1 is induced by hemin and a multiplicity of pro-oxidant stimuli including oxidative stress, inflammatory cytokines, heat shock, and ischemia-reperfusion (34, 37, 52). Therefore, the transcription of HO-1 can be activated by many stimuli. The HO-1 promoter has a plurality of different response elements, which bind the corresponding activated transcription factors [reviewed in Ref. (53)]. Therefore, the current literature suggests that the transcription factor activity of HO-1 through its multiple stimulators is indirectly regulated through activation of signaling cascades that are dependent on protein (de)phosphorylation, reduction-oxidation (redox) reactions, or both. A variety of protein kinases A and G, tyrosine kinases and the enzyme of the PI3K/AKT/Nrf2 (phosphatidylinositide 3-kinase, AKT, nuclear factor (erythroid-derived 2)-like 2) pathways are involved in the activation of HMOX1 (HO-1 gene) (54–56). Furthermore, HO-1 expression in macrophages was enhanced after LPS stimulation when PKA was additionally inhibited (57). As one of the principle mediators in this setting, the MAPK (mitogen-activated protein kinase) cascades seem to be involved. MAPKs are a ubiquitously expressed signal transduction supersystem that regulates countless cellular processes such as growth, motility, differentiation, and apoptosis. HO-1 inducers are hypothesized to activate the MAPKs. The MAPK supersystem consists of three subdivided primary signaling pathways that can then induce the following transcription factors: HSF1 (heat shock transcription factor), Nfr2 (nuclear factor erythroid 2-related factor 2), AP (activator protein)-1, and NFκB (nuclear factor). These transcription activators can then induce the HO-1 gene HMOX1 either directly or through an association with another DNA-binding protein (54). Still, this pathway is quite complex and much remains unknown. For example, some HO-1 activators such as cadmium activate all three MAPK pathways (58), while others are more specific (59). However, the activation of a particular MAPK does not automatically lead to HMOX1 induction, highlighting the complexity of the system.

Al-Huseini et al. described the signaling cascade for HO-1 in dendritic cells (60). They found that HO-1-regulated dendritic cell maturation and function in conditions of inflammation are mediated *via* p38-MAPK. As described above, this MAPK can again in turn induce the transcription of HO-1. Sepsis or LPS-induced autophagy prevented hepatocellular death in part *via* an HO-1 p38-MAPK signaling pathway (61).

The signaling cascades of adenosine are also quite complex and actually include several possibilities in different settings but adenosine has also been shown to act *via* multiple signaling pathways in the same setting. For example, Che et al. demonstrated that the A_2A_-receptor mediated increased collagen production in hepatic fibrosis *via* PKA and p38-MAPK (62). In Alzheimer’s disease, adenosine controls the neurotoxicity of the β-amyloid peptide *via* the p38-MAPK but not the PKA-pathway (63).

Therefore, the signaling pathways of HO-1 and adenosine intersect at PKA and MAPKs but are both still incompletely understood.

Our studies suggest that, in our model, HO-1 stimulation is not efficient if the adenosine receptor A_2A_ or A_2B_ is not expressed and that this might be regulated on a transcriptional level.

**Figure 7:**
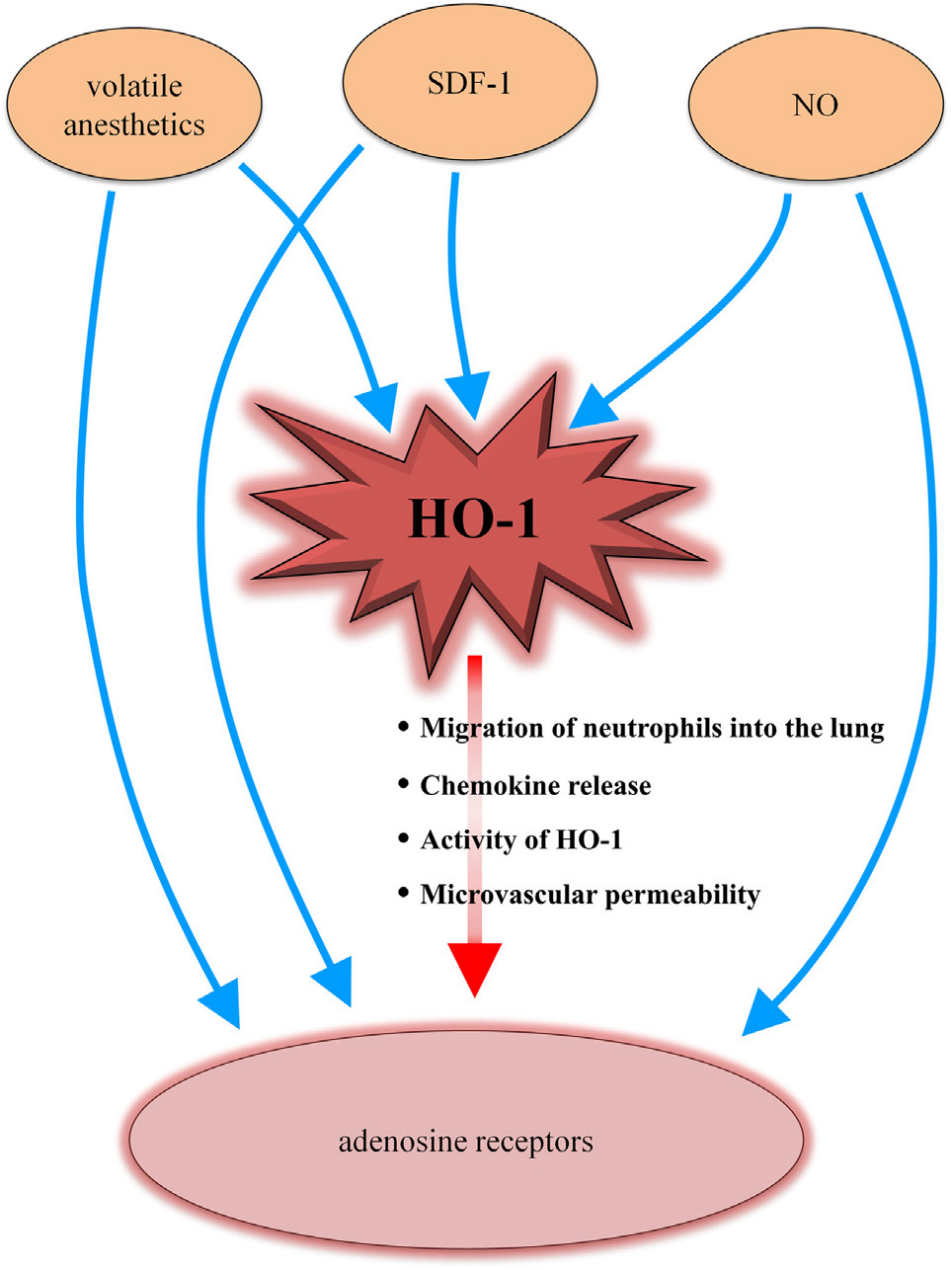
Heme oxygenase (HO)-1/adenosine receptor axis in the context of different inflammatory-mediated systems. Volatile anesthetics influence the HO-1 enzyme as well as adenosine receptors. The chemokine stromal cell-derived factor (SDF)-1 and nitric oxide (NO) have been shown to interact with HO-1 and adenosine receptors. Our data now complete the puzzle by adding a connection between HO-1 and adenosine receptors (red arrow), explaining the findings of several other studies.

Furthermore, the presented link between HO-1 and the adenosine receptors results in new insights on the interaction and connection of different systems involved in terms of inflammation (Figure 7).

Volatile anesthetics, such as sevoflurane or its precursor isoflurane, induce HO-1 (64, 65). Additionally, the adenosine receptors play a pivotal mechanistic role in terms of the volatile anesthetic-associated effects (66, 67). The chemokine stromal cell-derived factor (SDF)-1 induces HO-1 (68) and the anti-inflammatory effects of SDF-1 have been linked to adenosine receptor signaling (69). HO-1 expression has been shown to be modulated by nitric oxide (NO) (70). NO is connected to the adenosine pathway, which is further described in the ALANO pathway (adenosine/l-arginine/NO). Adenosine activates adenosine receptors, resulting in increased l-arginine transport and NO synthesis (71, 72). Our results now link these different studies together by presenting a new connection between HO-1 and the adenosine receptors.

The importance of the A_2A/2B_-dependent signaling for the anti-inflammatory effects of HO-1 further highlights the clinical importance of the presented study. Recent literature has demanded the identification of subgroups of patients to determine specific treatment strategies for acute pulmonary inflammation (1, 3). Since septic patients have altered adenosine receptor expression and ligand affinity (22), the adenosine receptor expression and ligand affinity of these patients should be explored before a therapy with hemin is considered.

## Conclusion

Our data link the anti-inflammatory effects of HO-1 to adenosine A_2A/2B_-receptor signaling and, therefore, may explain why targeting HO-1 in acute pulmonary inflammation has failed to prove effective in some patients. Furthermore, the correlation between HO-1 and adenosine receptor signaling provides a deeper insight into inflammatory signaling and represents a new correlation that can explain the link between other enzymatic/chemotactic settings under conditions of inflammation.

## Ethics Statement

All animal protocols were approved by the Animal Care and Use Committee of the University of Tübingen.

## Author Contributions

All authors made substantial contributions to this article. CZ, K-CN, and FK mainly contributed by participation in the data acquisition, analysis, and interpretation. FK and JR contributed to the conception and design of the study, as well as the analysis and interpretation of the data. FK and JR wrote the manuscript.

## Conflict of Interest Statement

The authors declare that the research was conducted in the absence of any commercial or financial relationships that could be construed as a potential conflict of interest.
