# Supplementary material for: Anti-inflammatory Effects of Heme Oxygenase-1 Depend on Adenosine A2A- and A2B-Receptor Signaling in Acute Pulmonary Inflammation
Source: Front Immunol. 2017 Dec 20;8:1874. doi: 10.3389/fimmu.2017.01874 (PMC5742329; doi:10.3389/fimmu.2017.01874)
Supplement: Supplementary file 1 [file Presentation_1.PDF]

Supplemental figure 1

A

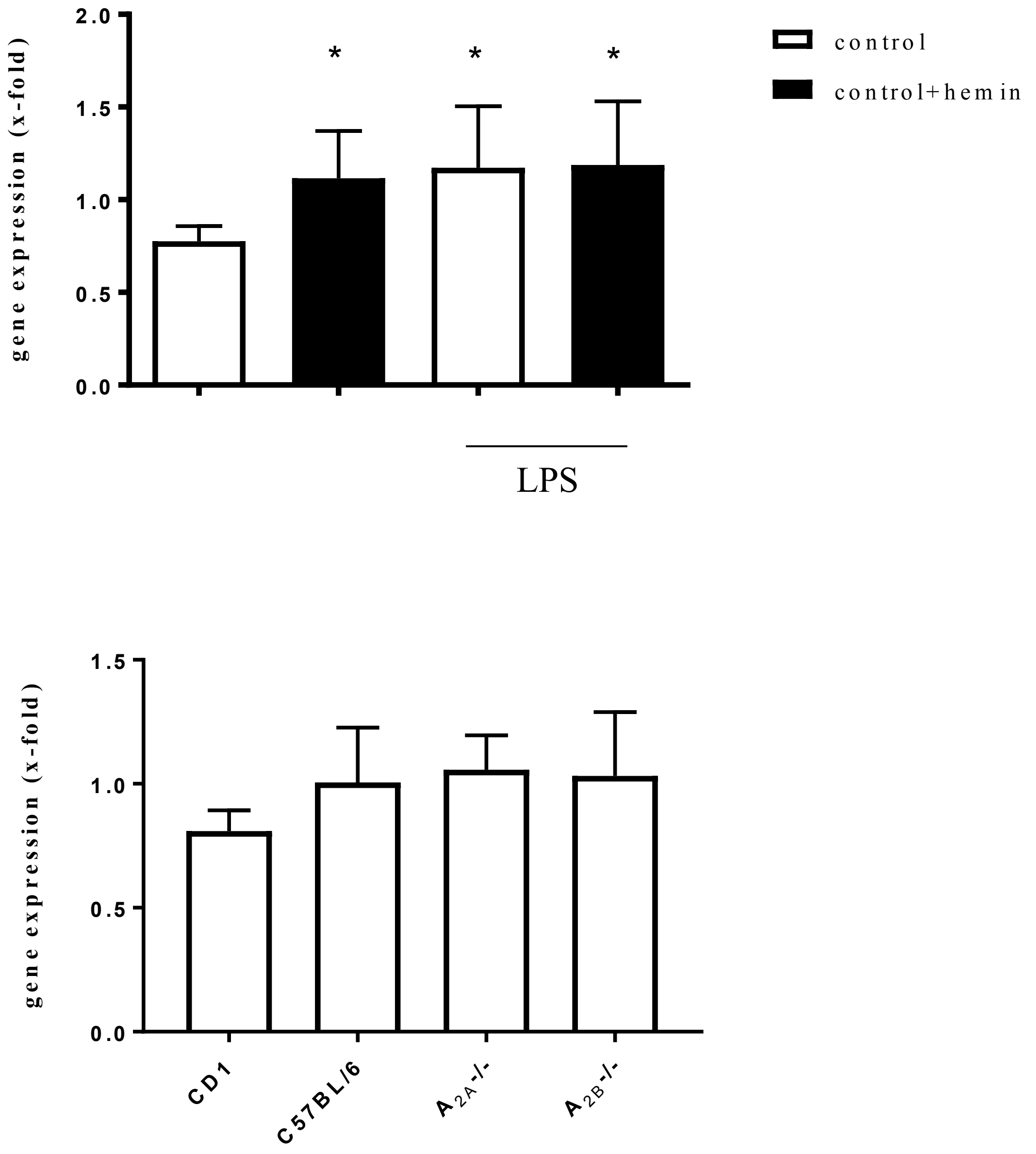

Supplemental figure 1

B

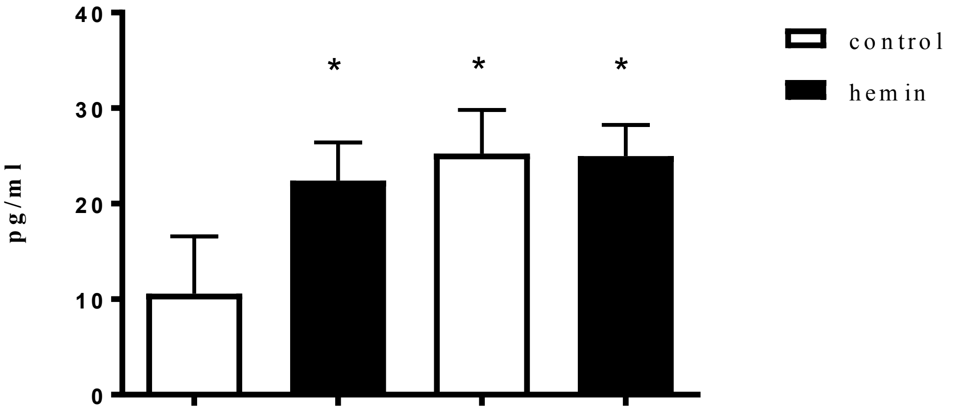

Supplemental figure 1

C

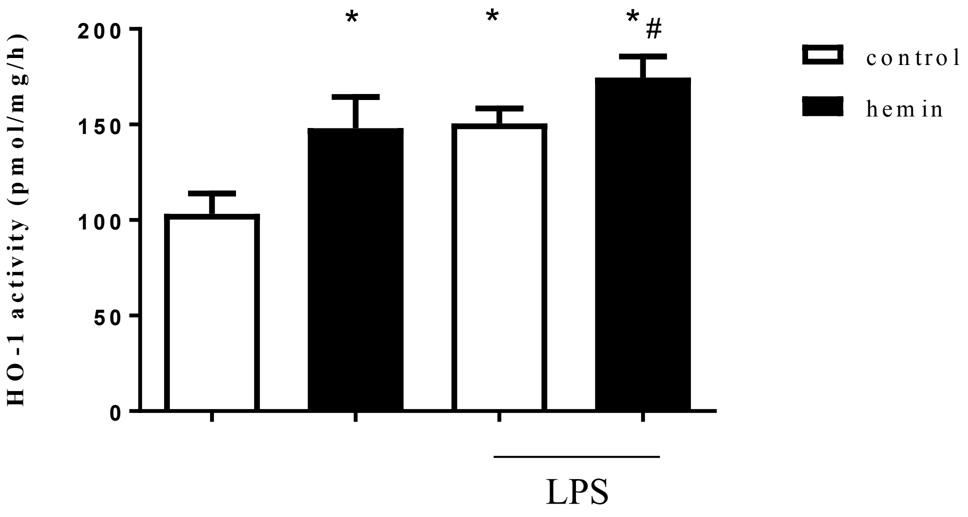

Supplemental figure 2

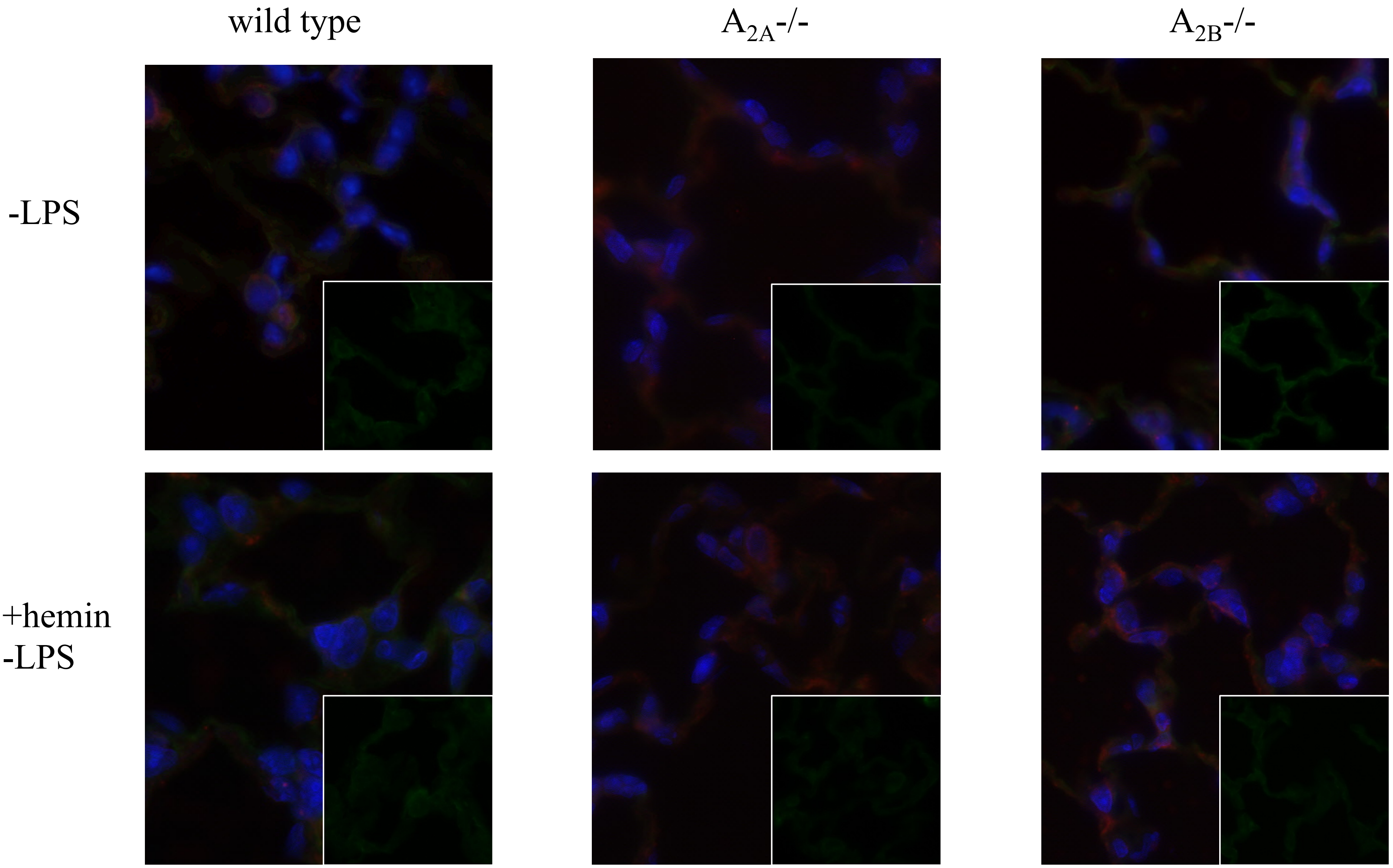

Supplemental figure 3

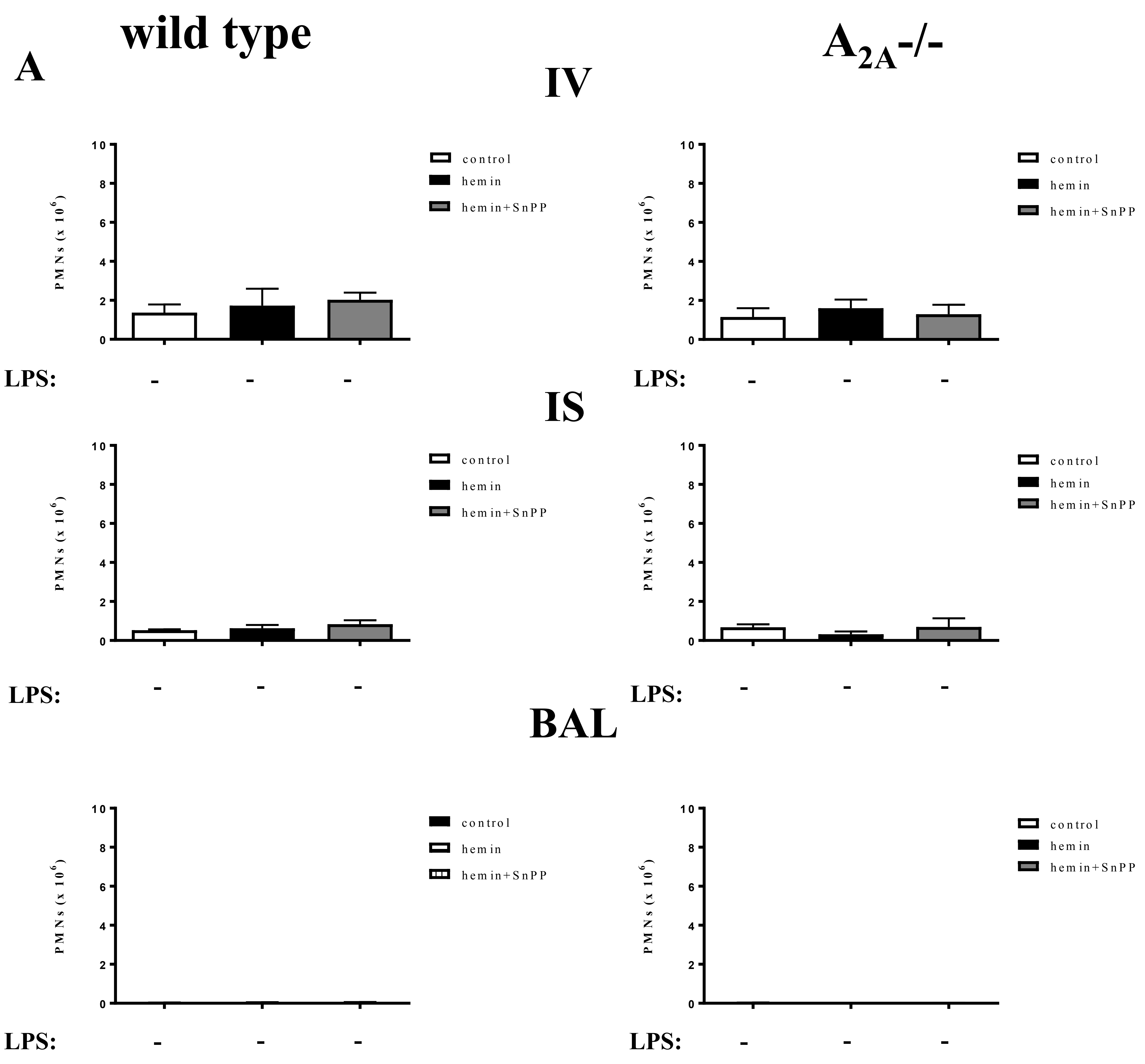

Supplemental figure 3

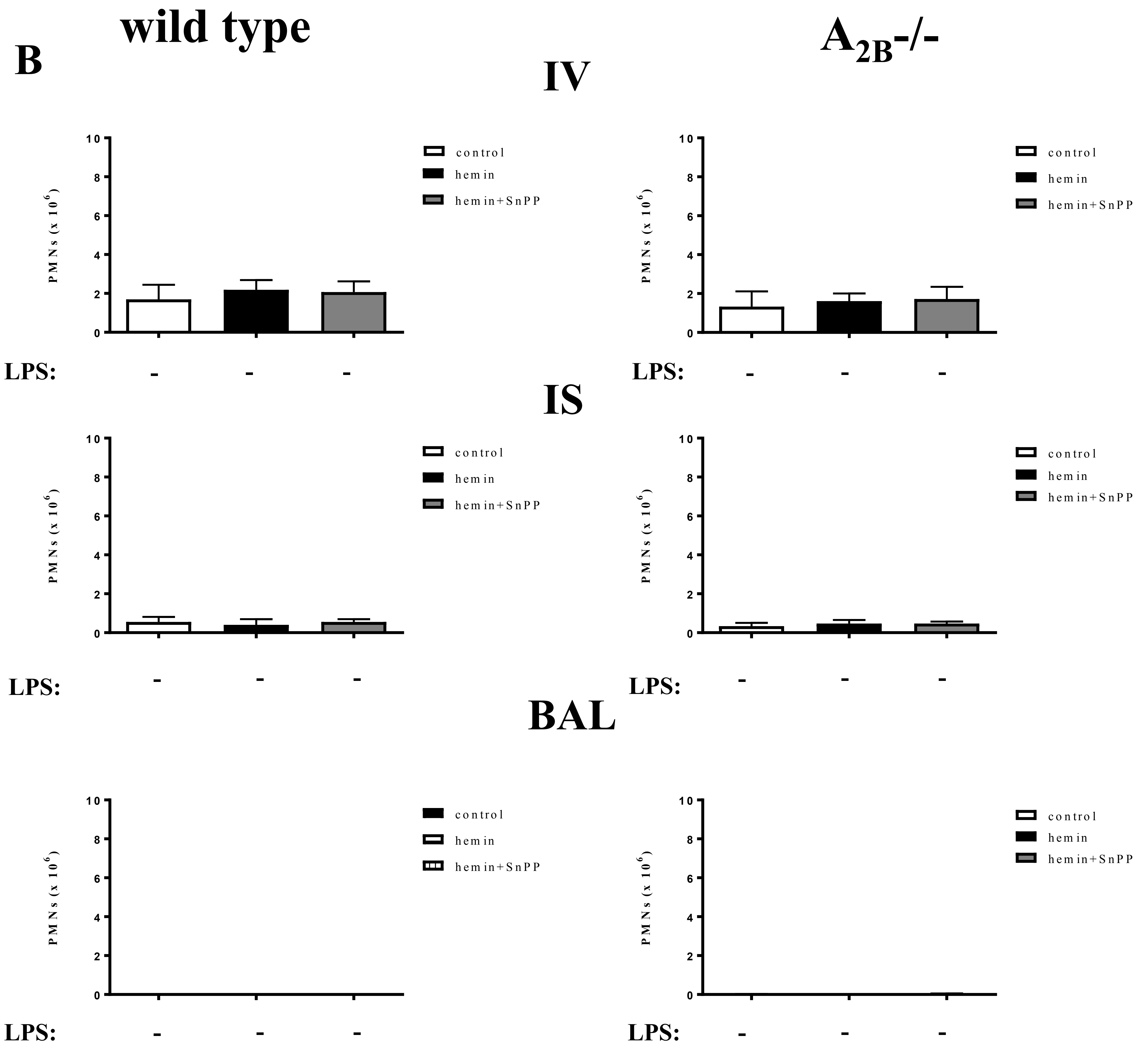

Supplemental figure 4

A

wild type

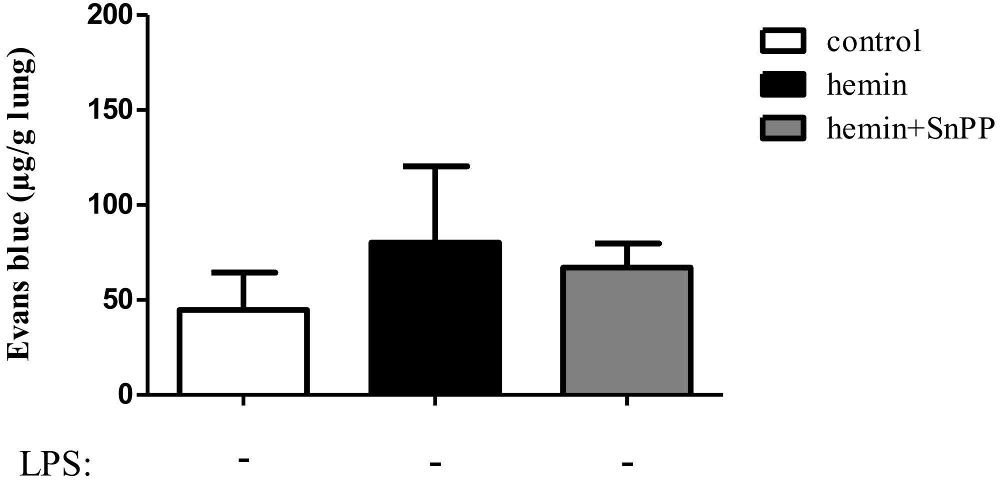

A_{2A}-/-

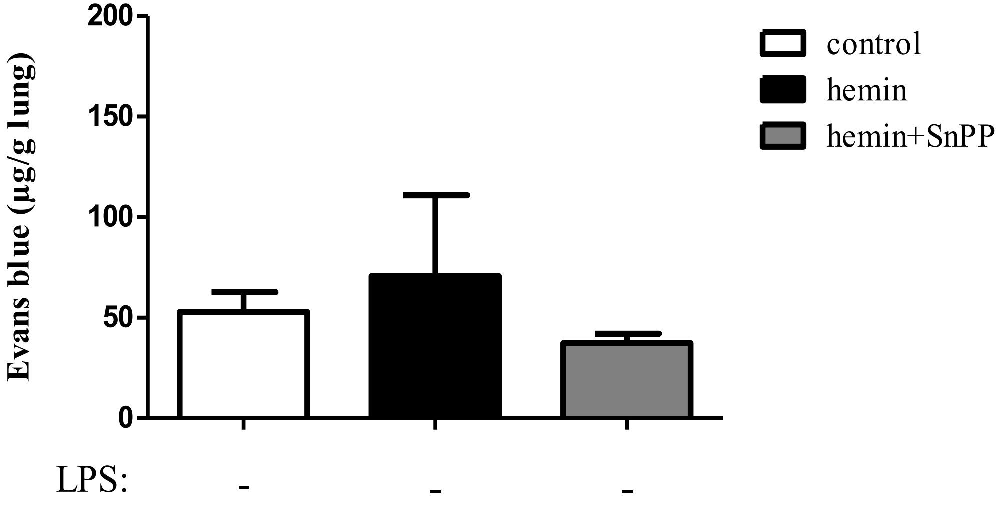

Supplemental figure 4

B

wild type

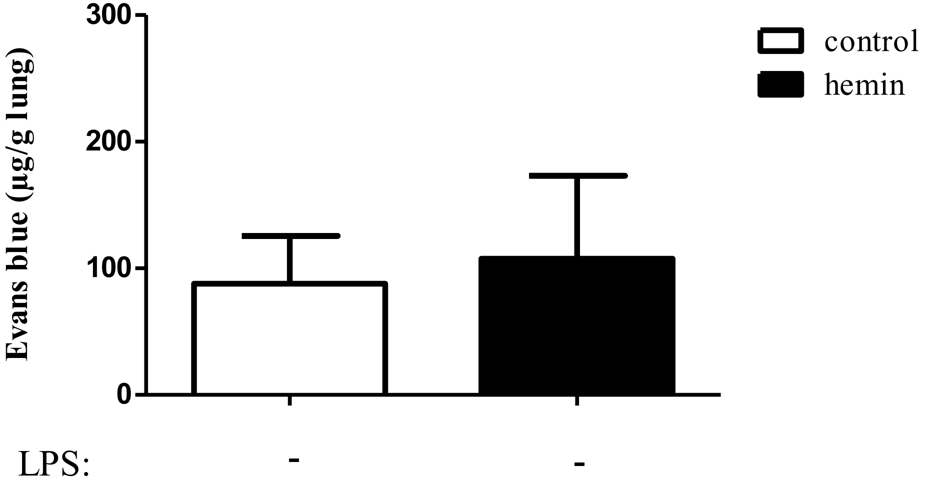

A_{2B}-/-

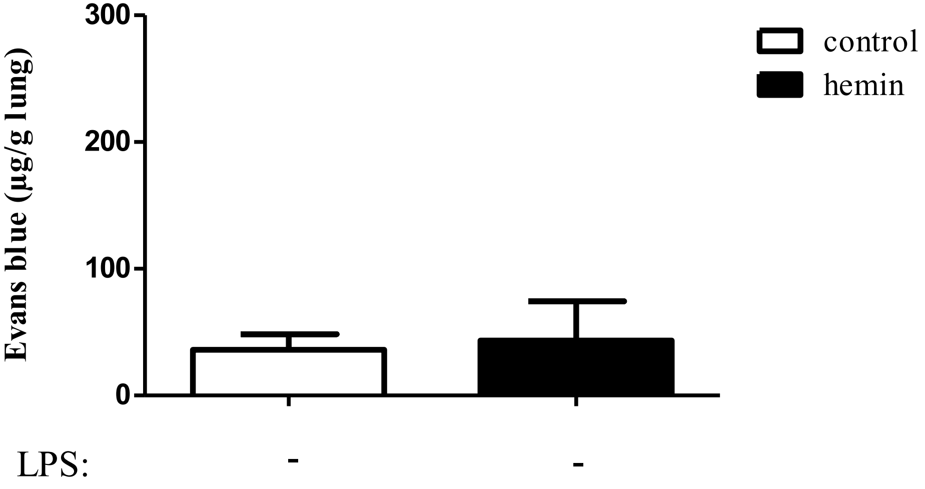

Supplemental figure 1: Gene and protein expression in C57BL/6 mice and in comparison of the different mice strains. Gene expression of HO-1 in C57BL/6 wild type mice (n=5-8) and the expression of HO-1 in all animal strains in comparison without inflammation (**A**). Protein expression of HO-1 (n=6) (**B**) and HO-1 activity (n=4 without LPS, other groups n=5-6) (**C**) in C57BL/6 wild type mice (n=5-8).

Supplemental figure 2: Determination of HO-1 expression in lungs of mice without inflammation by immunofluorescence. HO-1 was induced by hemin in wild type, A_{2A}-/- and A_{2B}-/- mice (n=4). Images are representatives of four experiments with similar results (original magnification, x63). HO-1 was stained with a specific antibody and appears green, nuclei were stained with DAPI and emerge blue, cytokeratin appears red.

Supplemental figure 3: Migration of polymorphonuclear neutrophils (PMNs) into the different compartments of the lung after activation of heme oxygenase (HO)-1 without inflammation. HO-1 was induced by hemin and inhibited by the additional administration of SnPP in wild type, A_{2A}-/- (**A**) and A_{2B}-/- mice (**B**). Migration of PMNs into the different compartments of the lung (IV=intravascular, IS=interstitial, BAL=bronchoalveolar) was evaluated. Data are presented as mean ± SD; without LPS-inhalation n=4.

Supplemental figure 4: Effects of HO-1 on microvascular permeability without inflammation. The capillary leakage was assessed by Evans blue extravasation and the influence of HO-1 in wild typ, A_{2A}-/- (**A**) and A_{2B}-/- (**B**) mice investigated. HO-1 was induced by hemin and inhibited by the additional administration of SnPP. Data are presented as mean ± SD; LPS-inhalation n≥4.
